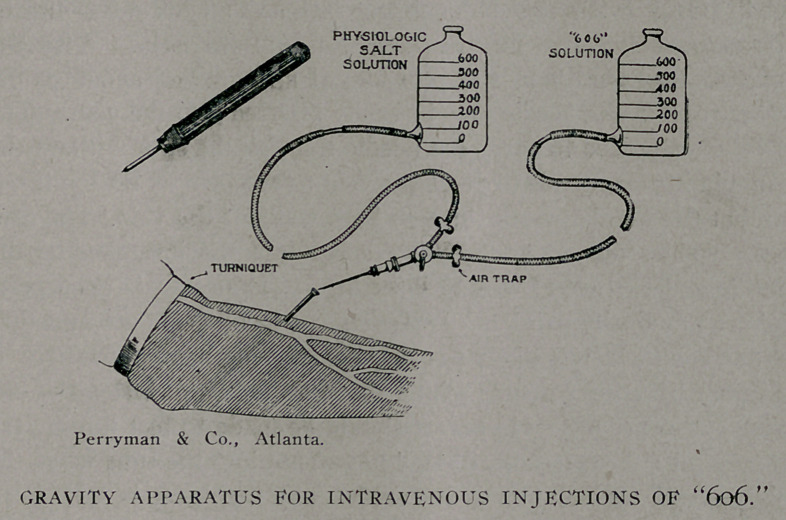# “606;” Its Administration 455 Times

**Published:** 1911-12

**Authors:** Edgar G. Ballenger, Omar F. Elder

**Affiliations:** Atlanta, Ga.; Atlanta, Ga.


### "606;" ITS ADMINISTRATION 455 TIMES.

By Edgar G. Ballenger, M. D. and Omar F. Elder, M. D.,
Atlanta, Ga.

The earlier reports on the use of "606" or salvarsan, were more or less incomplete, owing to the reason that they could not include facts regardng the permanency of the curative results. During the past ten months a very large number of patients have received "606" in this country and many reports concerning it have been made in Europe. Therefore, we can make at least a preliminary forecast of the results of this plan of treatment. While it will take many years for the ultimate cure to be proved, it now seems certain that in "606", properly administered, we have the best treatment for syphilis. It is the *best* in many ways:

First, it quickly eliminates the danger of spreading the disease to others, as nearly all observers report the prompt healing of syphilitic lesions.

Second, it is much less disagreeable to the patient than is treatment with mercury and iodide of potash.

Third, properly administered it seems less harmful than mercury and iodides.

Fourth, it cures very quickly affections that were uncured after prolonged courses of treatment with other remedies.

Fifth, it often works like a miracle in arresting the progress of malignant syphilis.

Sixth, patients gain in weight and strength to such an extent that those who used it properly are compelled to admit the superiority of "606," as well as its harmlessness.

Seventh, the fact that recurrences are very infrequent in the patients who received treatment 8 and 10 months ago and who received *only* this remedy, indicates that the curative results are to be much more lasting and complete than were ordinary courses of mercury.

Eighth being more pleasant to take, the patients are willing to repeat, if necessary, the injections and thus obtain a more thorough cure than many who depend alone on mercury and potassium iodide.

Ninth, if it is deemed necessary, the good produced by "606" may be supplemented by mercurial treatment, which in such instances does not require such large quantities of mercury as formerly, when this remedy alone was depended upon to effect the chief part of the cure, therefore properly used "606" should remove the harmful effects of mercury.

Tenth, mercury may be employed to clinch the prompt work of "606," or "606" used to supplement and complete the cure started by mercury.

Eleventh, the unusual interest now taken in the thorough treatment of syphilis ought to perceptibly lessen the number of patients who suffer from parasyphilitic and late nervous affection, which clearly come from uncured syphilis.

Twelfth, since *beginning* nervous affections are favorably influenced by "606," it does not seem unreasonable to hope that such affections may be prevented by its repeated injections until the Wassermann test remains negative.

Thirteenth, the scientific manner in which "606" was studied and principles upon which its discovery was based mark a distinct epoch in the treatment of disease and blazes the way through therapeutic nihilism perhaps to better cures of other diseases.

Fourteenth, substantial acknowledgement of certain life insurance companies as to the permanency of the cures by "606," is shown by the fact that they will now accept at ordinary rates the patients who have received treatment with "606" and whose blood test is found negative at three tests during a period of one year.

Our enthusiasm in the use of "606" has increased rather than diminished after having ministered 455 injections to about two-thirds that number of patients. From the start we were not an advocate of relying upon a single injection. There is no valid reason why subsequent injections *should not be administered,* as the prompt manner in which the recurrent lesions clear up after the second or third treatment shows that the spirochetes do not become immunized to "606" and that they are quite as promptly destroyed by subsequent injections as were those subjected to the influence of the first. The micropathology of syphilitic lesions shows that the spirochetes may become incapsulated in small blood or lymphatic vessels and connective tissue spaces, walled in, as it were, by nature in her effort to limit the spirochaetal invasion. When pocketed and protected in this manner the spirochetes may not be killed by the remedy, no matter how potent, circulating in the blood. The majority of the spirochetes are fortunately not thus protected and when destroyed the pathologic tissue is absorbed and the next administration of "606" (one month later) kills the remainder of the spirochetes or the majority of them. This fact seems proven by the scarcity of recurrences or positive Wassermann tests in patients who received this treatment 6 to 10 months ago. When recurrences do appear they are isolated as a rule—not widespread as they often are after the mercurial treatment. If all of the spirochetes are not destroyed by the injectons a return of the lesions will usually develop about 3 months after the last treatment and it is nearly always preceded by a positive blood reaction. We have, therefore, a fairly reliable test as to whether further treatment is indicated. If it is needed, which shall we use "606," or mercury, or both? Sufficient time has not yet elapsed after the administration of "606" to permit of hard and fast rules. Some suggestions may be made, however, which may be taken tentatively as a working basis. Since "606" has many advantages, when the administrator is so equipped that it may be given expeditiously intravenously, it would seem advisable at the present to depend upon a repetition of this remedy until a perfect cure has been effected. Intramuscular injections of mercury or inunctions should be administered to the small number of patients who may need the supplementary action of this drug to cure or render permanent the good work done by "606." Only those patients should be given mercury who *need it,* if they are under careful observation. The injection of "606" is always repeated at he end of a month or earlier and then we wait for a return of symptoms or a positive Wassermann for about three months. At this time recurrences seem most likely to develop. If then there is any doubt as to the condition or if the Wassermann is positive another intravenous injection is advised. This is preceded or followed occasionally by mercurial injections.

We do not like to start mercury until it is clearly seen that it is necessary for the reason that it masks the symptoms and may make us think the patient is in better condition than he really is. If the cure is not complete we wish to know it as soon as possible so that more "606" and a long course of mercury may then be given. More than 95 per cent. of our patients have remained well from 3 to 10 months without mercury or more "606." The remainder have quickly responded to more treatment and are now in splendid health, except one patient who received 4 intravenous injections of "606," salicylate of mercury intramuscularly, inunctions and iodide of potash without being cured of a large flat papular syphilide of the chest and body. This is the only instance in which we have not been able to effect a satisfactory cure of an active syphilitic process. We believe in the course of time this patient will be cured, when his general strength has been increased until he develops a better resistance.

The small number of recurrences seen in the patients who received two and three intravenous injections of "606," at properly spaced intervals, has been most gratifying, and we have no reason to doubt that those who have remained well and untreated for 6 to 10 months are not cured. The blood test is of value in determining if more treatment should be administered but should be disregarded when negative and not in accord with clinical facts.

Krefting has reported a well studied case of reinfection with syphilis after treatment with "606." Such an occurrence is thought to prove beyond doubt that the patient was cured absolutely. I have recently seen a patient who has contracted a chancre 4 1-2 months after an intravenous injection of "606." The first chancre came on in 1898, 3 weeks after intercourse and was diagnosed undoubted syphilis by Dr. F. W. McRae. Mercury and potassium iodide were taken for 18 months. The patient remained well until April, 1911, when a persistent soreness and redness developed in the nose and throat. This quickly subsided after an injection of "606;" the patient gained in weight and improved greatly in general health. Four and one-half weeks later an indurated sore developed after an incubation period of three weeks. Typical *spirochaetae pallidae* were demonstrated in the secretion from the lesion. This promptly healed after another injecton of "606." The rarity of second infections with syphilis when treated with mercury and potassium iodide and the frequency with which parasyphilitic affections of the brain and nervous system are seen would appear to indicate that in the past a very large number of syphilitics have not been *completely cured*. If they had been would our insane asylums be overflowing with patients, many of whom show positive blood tests? As much as we appreciate the value of mercury and iodide of potash, are we not seeing constantly its failures? Certainly a record of the application of mercury since the 15th century shows that it is *not sufficient*. We might answer that the remedy is all right, but that the patients have not taken it properly nor for a sufficiently long time. Here is where the rub comes. To cure completely the disease it is not infrequently necessary for the patient to take it so long that the disagreeable and harmful effects become so troublesome that the patient is willing to take a chance about the future in order to get relief from the ever present mercury. He goes to his physician for help, and more mercury and iodides are recommended. He goes to Hot Springs and more mercury is rubbed in. Finally he quits treatment to await parasyphilitic manifestations while he tries to regain his weight and recover from gastro-intestinal disturbances, salivation, and other troubles caused by the prolonged course of mercury. These difficulties *now* should be rarely seen for if he be so unfortunate as to require mercury to supplement "606" or to render permanent its healing effect, the quantity required is so much less than when it alone is used that the harmful effects may be considered trivial or non-existent.

The dangers of "606" properly administered are of small importance when compared with mercury or the disease. So far we have seen no disagreeable effects from "606" except occasional pain in our early cases where part of the medicine escaped subcutaneously, causing temporary pain. Fever, nausea, vomitting and diarrhoea the first night may be expected in the majority of patients with active acute symptoms. When the disease is just beginning or in a latent stage these symptoms do not occur in the majority of patients. The patient rarely complains of these symptoms, however, as they are as a rule mild and brief. Dozens of patients have stated that they preferred the ill effects of "606" to calomel. We have seen one patient who became unconscious 5 days after taking "606." While we have no positive proof as to what the cause was we are convinced it was some way produced by "606." The patient soon recovered from this coma. We have seen no patient develop optic atrophy or deafness following the administration of "606." Five were given this remedy who had optic atrophy before the treatment. This condition showed practically no improvement, probably because the disease was too well established with too much destruction of nerve tissue, before the treatment was given. Personally judging from more than 455 administrations of "606," we believe that this remedy properly administered intravenously will prevent a hundred patients from going blind where one will develop an atrophy from it, if any at all do. If given subcutaneously or intramuscularly and allowed to lie in an unabsorbed mass for months where oxidation or other changes could take place, the possibility of a dangerous arsenic product forming from "606" and injuring the eye or ear seems not unlikely. This, however, is not the fault of the remedy, but of its administration. Ehrlich has recently said that for months he has used all of his influence, both by word of mouth and pen to stop now and forever the intramuscular injection of "606." Besides causing the patient much pain, it is many times inadequate as it remains unabsorbed and thus may become a menace. The second and other injections may have to be withheld at a critical moment or indefinitely because of the danger of the cumulative action of such treatments. That splendid clinical results follow its use in this manner all admit, but it has so many *disadvantages* it is rarely indicated. Our tendency at present is not to increase the dosage but rather to give moderate doses at first and a little larger one at the second treatment. This gives quite a satisfactory cure and eliminates untoward effects.

Matson has recently made a report from Vienna where most of the unfavorable results and neurorecurrences have been seen. He states: "It is worthy to note that nearly all of the disturbances following '606' have been reported as occurring in the early stages of syphilis, between the second and eighth month after infection. This evidence is utilized by the supporters of '606' as proof of the syphilitic nature of these complications. In addition all cases have occurred after subcutaneous and intragluteal injections and few, if indeed any, after intravenous injections. This being the most intensive method is a further argument of Ehrlich's supporters that these effects are luetic in nature and not due to '606.' They furthermore claim that such disturbances are just as common after mercury. Prof. Finger admits the luetic nature of the cranial nerve disturbance but believes that '606' is related to it."

So far we have seen no neurorecurrences, probably because of the fact that all, except few injections early during the year, have been intravenous, and nearly always repeated at the end of a month ad again if indicated.

If the patients who received "606" had been selected by syphilographers, to be sure of the diagnosis; if the physical examinations, to determine their fitness, had been made by physicians accustomed to making such examinations; if the remedy had been prepared by exact chemists; and if the preparation and administrations had been done by persons with a perfect surgical cleanliness and if surgeons, expert in blood vessel work, had administered intravenous injections, repeated them one month later, and again, if the uncured disease or a positive blood test showed them to be indicated; if all of these *ifs* had been observed by all of the administrators of "606," it would have received nothing but praise for its *wonderful action* and for its *harmlessness*. We must remember that "606" has been passing through the experimental stage, during which much information has been gained. Many patients have also been treated by men entirely unfamiliar with its exact preparation and mode of administration. Even during these circumstances, with *far too much* of it given subcutaneously and intramuscularly, *very few* ill effects have been recorded compared to what might have been expected. These facts prove most conclusively that properly administered it cannot be considered a dangerous remedy. Facts have been piled on facts to prove that it is our most potent remedy in killing spirochetes and curing syphilis. No drug has ever been subjected to such careful study of its curative and after effects as has "606" and yet those who have administered it most assiduously are its most enthusiastic advocates. These men have developed a technic in the diagnosis, physical examination, preparation and administration of this remedy that has enabled them to combine the before-mentioned requirements and in the future they will be able to show clearly the difference between the results after the proper and the improper administration of which one symptom is so exaggerated as to dwarf, or even de- "606." Many of the unfavorable reports have come from beginners; its misuse should not properly be blamed upon the remedy itself. Under the circumstances it is remarkable that such a potent remedy could have been administered to more than 200,000 patients without more harm, especially as the remedy was new and no technic was perfect at the start.

Only after the physician has weighed the advantages and disadvantages of "606" against an uncertain, tedious and perhaps harmful course of mercury and the well recognized dangers of syphilis, is he competent to give correct advice to his patient.

In Surinam there have been altogether nine hundred framboesia patients treated and only three haave suffered a relapse. In the framboesia hospital at Groningen, in Surinam, there were 328 patients suffering with framboesia. The seven physicians in charge and five medical students administered salvarsan to all of the patients in the course of eight days; after two weeks the last patient was dismissed from the hospital and thus there appears perhaps the only recorded case in the history of medicine in which the hospital erected for the treatment of a malady had to be closed.—(Ehrlich.)

Leissl (Berliner klin. Wochenshrift, Nov. 6, 1911), has reviewed Schmidt's year-books from 1845 to 1904 and found summaries of articles from nineteen authors describing from one to twenty-five cases of syphilitic affections of the optic or auditory nerves and a compilation by L. Gros and Lancereaux in 1861 of neuroses, induced indirectly by changes in the surroundings of the nerves. Even from this incomplete reviews, he says, the frequency of nerve trouble in the course of syphilis long before the discovery of "606" is established beyond question.

### Technique of Intravenous Injections.

The majority of investigators now believe that the best method of administering salvarsan is by the intravenous method; that this view is correct, we are confident, having discontinued all other methods of giving it ten months ago. Those who have administered many intravenous injections have at times experienced difficulty in inserting the needle directly into the vein, without a previous incision through the skin. As many patients receiving

Perryman & Co., Atlanta.

GRAVITY APPARATUS FOR INTRAVENOUS INJECTIONS OF "606."

an incision, through which the needle was passed into the vein, were complaining of the tell-tale scars that remained, we endeavored to devise a method whereby all patients might receive the intravenous injection without the incision and herewith submit a simple plan which, in our hands, has greatly facilitated the insertion of the needle.

To begin with everything should be done with surgical cleanliness. The patient's arm, usually the left, is prepared for four inches above and below the elbow. Any of the ordinary preparations are sufficient. We paint the area with two coats of iodine and find it a very easy and effective method of skin sterilization.

After the injection the iodine may be removed with 10% ammonia water. The patient reclines and holds the arm up while it is wrapped above and below the sterilized portion with sterile towels; it is then placed on a table covered with sterilized cloths. A turniquet is adjusted over the towel around the arm so as to make the veins stand out prominently. The largest one near the elbow is selected and a few drops of a 2% solution of cocaine are injected into the skin over the vein and into the subcutaneous tissue around it. A point just over the vein is noted and, while the skin is pulled aside in order not to puncture the vein, a small trocar is inserted through the skin so that the distal end of the cannula lies just between the skin and the vein. With the cannula now held over the vein and at an angle of about 30 degrees from it, the needle may be easily inserted into the vein. The pressure required to carry the needle through the skin without the cannula in place tends to press the vein to one side or flatten it out so that the needle passes through both walls of the vein. The cannula enables one to gauge more accurately the insertion of the needle and further efforts at inserting it, if the first is unsuccessful are practically painless. The escape of blood from the puncture of the vein, which is likely to cause a confusing hematoma, may be lessened by using a needle with a sharp, round point instead of one with cutting edges. No effort should be made to test the inward flow until a free stream of blood passes out into the tube when the physiologic salt solution is lowered. The turniquet is now loosened, the solution is raised about three feet above the patient's arm and if it flows freely into the vein without a subcutaneous swelling the cock may be so adjusted as to allow the flow of the "606" solution, which with the same elevation flows into the vein slowly by gravity. The only dressing necessary after such a treatment is a small bit of collodion over the punctured wound. Occasionally the individual may be too fat to find a suitable vein or the veins may be so small that an incision from the first seems necessary. It is advisable not to prolong unduly the trials by salvarsan as it is eliminated, and thus prevent irritation of the the puncture method, but to make a small incision and expose the vein rather than to annoy the patient by tedious, unsuccessful attempts. The method just described undoubtedly facilitates the insertion of the needle and will lessen the number who require an incision to secure a proper flow, in fact, the writers have not found it necessary to make an incision in more than 1% of our last three hundred administrations. The site of the injection should be watched for swelling, as a subcutaneous leak may occur toward the end of the injection even when it starts perfectly. A burning pain is also felt by the patient if there is a leak. If any untoward symptoms should develop during an injection the treatment should be discontinued. So far in our own expedience no symptoms have occurred that prevented the patients taking the full amount of "606" indicated nor have the after effects of the treatment at any time seemed serious. An effort has been made to regulate *always* the dose to suit the *physical condition* and weight of the patient. The freedom from trouble so far experienced was probably due to careful physical examinations of all patients and a regulation of the dose to suit the condition and weight of each individual. We have also insisted that all patients drink freely of plain water and lithia water the day of the treatment and for two weeks afterward, in order to dilute well the kidneys.

Until a further test of the permanency of the "606" treatment has been made it would seem advisable that mercury in moderate amount be recommended for those patients who cannot report at frequent intervals for examinations by a syphilographer, or have the Wassermann test made at stated intervals. The patient should be treated until permanently cured and until the blood test remains permanently negative.

The ultimate success of treatment depends much upon the financial arrangement made between the patient and his physicians. If a definite charge by the syphilographer covers the course of treatment with "606" and mercury, until the patient is entirely cured, with this fact proven by the time test, and no other charge is made for inspection or treatment—except for the actual cost of the injection—patients will return much more regularly and thus will obtain much more thorough cures than will the patients who pay a fixed charge for each injection. As the amount of medication, when "606" is administered, is so slight and as we are much interested in our failures and anxious to have them return, such an agreement can be made at a moderate price—usually more moderate than when mercury alone is given because less time and trouble are required to effect the cure. As the patient has paid for further observation and treatment he is willing to return for it and does not depend alone upon his own feeling of well-being as an index of his condition. If the administrator of "606" is not accessible, the importance of further careful observation and treatment should be impressed upon the patient and he should be urged to pay in advance, if convenient, or to make definite arrangement for its payment, a reasonable fee to his physician, for at least two years of observation and supplementary treatment when necessary; at the present it should be deemed necessary where one skilled in the management of syphilis is not readily available and in charge of the patient making examinations and taking blood at suitable times for the Wassermann test.

The patient usually sees such decided improvement in his physical and mental condition that unless he has paid for it previously he does not think it worth while to take further treatment and thus may he develop a relapse which more attention and treatment could easily have forestalled.

We know of no one factor which will contribute more to the successful management and cure of syphilis than the above suggestion as to definite remuneration of both the syphilographer and the physician. Past experience has shown that better results follow when the patient knows he has paid for his treatment because he comes more readily for it. If he has not made definite plan he is likely to shift from doctor to doctor, and in this manner may miss the value of consecutive treatment and observation. Sometimes, too, the patient may be almost cured and then go to another physician who perhaps doubts the correctness of the diagnosis and discontinues treatment until a recurrence may develop.

During this period much valuable time is lost and perhaps a parasyphilitic or nervous affection arises.

In closing, attention is called to a paragraph from a recent paper by Holland, of Hot Springs:

"As salvarsan loses its halo and becomes more and more of a real drug, it inspires us with more confidence, as after all we want a reason for things and are much better satisfied with the material than with the miraculous. We are learning better the probable effects of the drug with its limitations and contra-indications so that we can rely on its action more and more in a rational way. As soon as salvarsan recovers from its ill-advised notoriety and publicity, and is used the same as would be quinine in malaria it will be less criticised and its real usefulness will become apparent. * * A fair trial is all that salvarsan requires to establish itself as one of our safest and most efficient specifics."

"We believe in it for most cases of syphilis and are a great deal firmer in our belief after having discovered its imperfections than we were when it was first put on the market."

## Figures and Tables

**Figure f1:**